# II Italian intersociety consensus statement on antithrombotic prophylaxis in orthopaedics and traumatology: Arthroscopy, traumatology, leg immobilization, minor orthopaedic procedures and spine surgery

**DOI:** 10.1007/s10195-012-0214-y

**Published:** 2012-12-07

**Authors:** F. Randelli, E. Romanini, F. Biggi, G. Danelli, G. Della Rocca, N. R. Laurora, D. Imberti, G. Palareti, D. Prisco

**Affiliations:** 1 Dipartimento di Ortopedia e Traumatologia V, I.R.C.C.S. Policlinico San Donato, S. Donato Milanese, MI Italy; 2 Divisione di Ortopedia e Traumatologia, Casa di Cura San Feliciano, Rome, Italy; 3 UOA Ortopedia e Traumatologia, Ospedale S. Martino, Belluno, Italy; 4 UO Anestesia Analgesia e Medicina Perioperatoria, Azienda Istituti Ospitalieri di Cremona, Cremona, Italy; 5 Anestesia e Rianimazione, Università degli Studi di Udine, Udine, Italy; 6 UOC Cure Primarie, ULSS 12, Venezia, Italy; 7 UO di Medicina Interna, Centro Emostasi e Trombosi, Ospedale Civile di Piacenza, Piacenza, Italy; 8 UO Angiologia e Malattie della Coagulazione, Azienda Ospedaliera Universitaria S. Orsola-Malpighi, Bologna, Italy; 9 SOD Patologia Medica, Azienda Ospedaliera Universitaria Careggi, Florence, Italy

**Keywords:** Venous thromboembolism prevention, Anticoagulant prophylaxis, Mechanical prophylaxis, Bleeding risk, Arthroscopic surgery, Trauma surgery, Non-surgical traumatology

## Abstract

Pharmacological prophylaxis for preventing venous thromboembolism (VTE) is a worldwide established procedure in hip and knee replacement surgery, as well as in the treatment of femoral neck fractures, but few data exist in other fields of orthopaedics and traumatology. Thus, no guidelines or recommendations are available in the literature except for a limited number of weak statements about knee arthroscopy and lower limb fractures. In any case, none of them are a multidisciplinary effort as the one here presented. The Italian Society for Studies on Haemostasis and Thrombosis (SISET), the Italian Society of Orthopaedics and Traumatology (SIOT), the Association of Orthopaedic Traumatology of Italian Hospitals (OTODI), together with the Italian Society of Anesthesia, Analgesia, Resuscitation and Intensive Care (SIAARTI) and the Italian Society of General Medicine (SIMG) have set down easy and quick suggestions for VTE prophylaxis in a number of surgical conditions for which only scarce evidence is available. This inter-society consensus statement aims at simplifying the approach to VTE prophylaxis in the single patient with the goal to improve its clinical application.

## Introduction

Venous thromboembolism (VTE) has a significant clinical and social impact due to its high incidence and severe possible sequelae. Pulmonary embolism (PE), with or without concomitant measurable deep vein thrombosis (DVT), is the direct cause of roughly 10 % of hospital deaths [1]. Although anticoagulant prophylaxis for VTE has been routine practice for a long time, the literature on the subject is by no means comprehensive and unequivocal, especially in orthopaedic and trauma surgery. This prompted SISET (the Italian Association for the Study of Haemostasis and Thrombosis), SIOT (the Italian Association of Orthopaedics and Traumatology), OTODI (the Italian Association of Hospital Orthopaedics and Traumatology) and SIAARTI (the Italian Society of Anaesthesia, Analgesia, Resuscitation and Intensive Care) to set up a working group in 2009 to define an inter-association consensus statement providing practical recommendations for the daily management of VTE prophylaxis in hip and knee replacement surgeries and the treatment of femoral neck fractures.

The publication and success of this consensus document [2] led to the demand for a similar document regarding VTE prophylaxis in the remaining major orthopaedic surgeries, the so-called minor orthopaedic surgeries and orthopaedic trauma. A scarce and low level of evidence is available in literature for those orthopaedic daily life topics. Furthermore, they are only partially and superficially approached in guidelines. This new consensus statement was, therefore, drawn up to provide a comprehensive series of practical and easily applicable advices to further widespread good clinical practice in the field.

As a substantial number of patients, who are the object of this consensus, are not hospitalized, representatives from SIMG (the Italian Society of General Medicine) were invited to join the working group, due to the key role of general practitioners in their continuity of care.

## Methods

Although best-clinical-practice guidelines are a fundamental tool for health care providers, there are many important fields in which the scarcity of the literature does not allow strong evidence-based recommendations to be made. This is the case for VTE prophylaxis in a significant part of orthopaedics and traumatology. Indeed, although the existing guidelines (ACCP [1] and NICE [3]) have been recently revised and updated by authoritative working groups using rigorous scientific method, they are complex documents that are not particularly clinician-friendly. Furthermore, the chapters on musculoskeletal pathologies and orthopaedic surgery cover only a small number of the wide range of pathologies and treatments that clinicians have to manage on a daily basis. This is an accurate reflection of the international literature as a whole, which features an abundance of information on certain topics and virtually none on others [4]. The solution to this problem adopted by NICE and ACCP was, in brief, to supply the guidelines that can be backed up by scientific evidence, and to ignore the rest. Though this approach is not questionable from a formal perspective, it is, however, lacking from a clinician’s point of view. Hence, drawing on the pragmatic spirit that inspired the first publication of the consensus statement on antithrombotic prophylaxis in hip and knee replacement surgeries and femoral head fractures [2], the intersociety working group decided to fill the void and produce a document more useful for clinical practitioners, as the previous one.

A work plan was set up to respond to the practical needs of the clinicians, allocating the orthopaedic and traumatic pathologies to simple but workable categories. The literature regarding each of these categories was analysed from a practical point of view, using advanced but non-coercive methods, in order to provide as large a pool of relevant information as possible. The sources thereby obtained were then processed and summarized prior to screening by the working group in a series of plenary sessions, until a consensus was obtained.

In certain cases the process was greatly facilitated by the above mentioned NICE [3] and ACCP [1] guidelines, whereas in others it was a more arduous task. Nonetheless, consensus was reached in each case, following open debate giving the appropriate weight to literature reports, clinical experience and contextual clues. As the working group is fully aware of the limitations of such an approach, the authors decided to avoid making true ‘recommendations’, instead limiting themselves to inviting the clinician to consider prophylaxis or not in each particular case. Despite the impossibility of providing firm, evidence-based guidelines, the group was convinced of the necessity of a reference text on these issues, and therefore, set out to include the current state-of-the-art in a document that is in effect a compromise between methodological rigour and clinical pragmatism.

## Thrombotic risk

The risk of VTE associated with surgery or orthopaedic trauma is conditioned by the interaction of two types of factors: (a) individual risk factors, linked to the conditions and characteristics of the patients themselves, and (b) treatment-related factors, arising from the specific features and consequences of the surgical or non-surgical procedure employed.

### Patient-related risk factors [5–11]

There are numerous patient-related risk factors (Table 1), which act through various pathogenic mechanisms that contribute to the generation of venous stasis and/or increase a state of hypercoagulability. The risk of venous stasis increases with age (due to decreased motor activity), obesity, immobilization (transitory or permanent) or confinement to bed for several days, trauma, application of plaster casts, and varicose veins. Numerous patient-related risk factors, whether congenital or acquired, transient or permanent, promote hypercoagulability, and consequently increase the risk of thrombosis.

**Table 1:** Individual risk factors that could suggest a pharmacological VTE prophylaxis

Risk factor	
Individual or family history of VTE (first degree relatives)	XX
Known congenital or acquired thrombophilia (Table 2)	XX
Active cancer or cancer treatment	XX
Obesity (BMI >30)	XX
Bed confinement (>3 days)	XX
Impediment to normal ambulation (weight bearing <10–20 kg, lower extremity immobilisation)	X
Age (>60–70 years)	X
Oestrogen contraceptive therapy or hormone replacement surgery (ongoing or within 1 month after suspension)	X
Pregnancy or puerperium (6 weeks after delivery)	X
Recent acute myocardial infarction (AMI) or stroke	X
Chronic heart failure	X
Chronic respiratory failure	X
Inflammatory bowel disease	X
Sepsis or severe infections	X
Large varicose veins	X
Nephrotic syndrome	X

*XX* factors associated with a high risk, *X* factors associated with a moderate risk

An established significant risk factor is a past history of VTE, whether idiopathic or stimulated by a specific trigger. Familial history of VTE should also be considered as a risk factor. Aging is also a favourable condition for VTE, as it is associated with increased blood coagulability. Likewise, the presence of one or more thrombophilic conditions (congenital or acquired, see Table 2), today well documented and clinically significant, involves an increased risk. While this is not a call for screening general population or pre-operative patients for thrombophilic condition, clinicians should take particularly care assessing anamnestic and known risk factors.

**Table 2:** Thrombophilic conditions (congenital or acquired) of clinical relevance

Thrombophilic condition
Congenital
Antithrombin deficiency
Protein C deficiency
Protein S deficiency
R506Q (Leiden) factor V mutation
G20210A prothrombin mutation
Acquired
Lupus anticoagulant syndrome (LAC)
Presence of antiphospholipid antibodies Anticardiolipin and/or Anti-beta_2_glycoprotein I

Several physiological states, such as pregnancy and puerperium or iatrogenic conditions, like oral contraceptives or hormone replacement therapy (HRT), are well known thrombosis risk factors, and may, alone, justify VTE prophylaxis. If a woman assuming oral contraceptives or HRT is scheduled for surgery, it would be wise to suspend the therapy at least 1 month prior to the operation. In case of an urgent need of surgery, she should immediately suspend those medications and be considered at a higher risk for thrombosis.

Finally, an extremely significant risk factor for thrombosis is the presence of diseases such as neoplasms, heart failure, respiratory failure, inflammatory bowel disease, nephrotic syndrome, sepsis, or a recent history of myocardial infarction or stroke. In all these cases, therefore, prophylaxis should be considered.

### Treatment-related risk factors [12–15]

In orthopaedic surgery, the risk of thrombosis, and therefore, the need for prophylaxis, can generally be considered as proportional to the duration and invasiveness (trauma, demolition) of the surgical procedure. Other surgery-related factors that may influence the likelihood of thrombogenesis are: the position of the patient on the operating table (particularly if prone), any forced twisting or traction of a limb that could damage the blood vessels, and the use of additional medical devices (in particular, the tourniquet). One significant thrombotic risk factor in both surgical and non-surgical interventions (lower limb plaster casts, brace, splints or appliance) is the length of time before the patient returns to normal ambulation. In fact, compression on the plantar venous plexus brought about by normal ambulation, and the concomitant activation of the calf muscle pump, are key factors to ensuring the centripetal venous return. Ambulatory patients with fully functional feet and ankle movements are, therefore, far less likely to develop venous stasis. Hence, as venous stasis can cause thrombogenesis (particularly in the calf), the need for antithrombotic prophylaxis should be evaluated in cases of impediment to normal ambulation and calf muscle function (ankle/calf splinting or casting and/or non-weight bearing, NWB). Although the precise contribution of plantar venous plexus compression and calf muscle pump function to the circulation has not been evaluated, and firm evidence is, therefore, lacking, a load of 10–20 kg should be considered the minimum for its activation.

## Haemorrhagic risk

Pharmacological VTE prophylaxis is based on the use of anticoagulants, whose use is limited in patients at high risk of haemorrhage, where inhibition of the physiological mechanisms responsible for the regulation of thrombus formation can trigger or worse bleeding. Unlike the more precise and individual stratification of thrombotic risk, even in the latest guidelines (for example, ACCP [1] and NICE [3]) the definition of haemorrhagic risk is limited to mere suggestions, ‘empirical’ recommendations, frequently not supported by clinical trials. Contraindications to pharmacological prophylaxis for VTE are reported as absolute or relative, but even in the definition of the absolute recommendations there is some discrepancy between different sources. For example, according to NICE [3] guidelines, a platelet count of <20,000/μL is an absolute contraindication, whereas in other statements the thrombocytopenia cut-off point is higher, at <50,000/μL. Likewise, there is considerable disparity in the information sheets provided with drugs used in VTE prophylaxis (LMWH and fondaparinux, for instance), particularly between the respective manufacturers’ warnings and absolute contraindications. These information sheets also tend to feature vague terms such as ‘clinically relevant bleeding’ or ‘lesions at risk of bleeding’, and therefore the decision of whether or not to begin prophylaxis needs to be made on a patient-by-patient basis, based on the clinician’s evaluation of the haemorrhagic risk versus the thrombotic risk.

### Absolute contraindications [1, 3, 16, 17]

- Active bleeding
- Untreated congenital coagulopathies (haemophilia and severe von Willebrand disease)

### High haemorrhagic risk factors (decisions on an individual case basis) [1, 3, 16, 17]

- Individual or family history of major haemorrhage
- Acquired coagulopathies (e.g., hepatic insufficiency with abnormal coagulation test results and/or platelet count)
- PT ratio or PT-INR >1.5
- APTT >1.25 (except in cases with antiphospholipid antibodies and no history of haemorrhage)
- Thrombocytopenia (<50,000/μL)
- Severe renal failure (creatinine clearance <30 mL/min)
- Cerebral metastases or cerebral angioma at risk of bleeding (confirmed by CT angiography or MRI)
- Recent haemorrhagic stroke or ischemic stroke (24 h)
- Gastric and/or genitourinary or ocular haemorrhage within the previous 2 weeks
- Medicines acting on haemostasis (e.g., anti-platelet, anti-inflammatory drugs)
- III degree arterial hypertension (230/120 mmHg)
- Acute infectious endocarditis (except that due to mechanical prostheses)

Wherever possible and indicated, the haemostatic defect should be corrected via transfusion or pharmacological means; severely hypertensive patients should receive the appropriate treatment, and the risk/benefit ratio of suspending anti-platelet or anti-inflammatory drugs should be evaluated. In cases of high haemorrhagic risk, mechanical and/or pharmacological prophylaxis (assessing the need to reduce dosage and/or start the administration only post-surgery) can be considered. When the high risk is transient, antithrombotic prophylaxis should be started as soon as the haemorrhagic risk is under control, and continued until the risk of thrombosis persists.

### Blood tests essential for assessing the degree of haemorrhagic risk

- Complete blood count to obtain:
  - Prothrombin time (PT)
  - Activated partial thromboplastin time (APTT)

- Coagulation screen to determine:
  - Platelet count
  - Haemoglobin concentration

### Basic rules

- In all patients needing pharmacological antithrombotic prophylaxis, it is advisable to evaluate both the thrombotic risk and the haemorrhagic risk, identifying patients at high risk and those who will need careful evaluation.
- In patients who cannot be prescribed pharmacological prophylaxis, it is advisable to use mechanical devices such as graduated compression stockings (GCS) or, in cases of high thrombotic risk, intermittent pneumatic compression (IPC) or plantar venous pump (PVP).
- When the contraindication is temporary, it is advisable to start pharmacological antithrombotic prophylaxis as soon as the haemorrhagic risk is under control, for as long as the thrombotic risk persists.

## VTE prophylaxis

In minor orthopaedic and trauma cases, VTE prophylaxis can be pharmacological (LMWH), mechanical (either active, i.e., IPC and PVP, or passive, i.e., GCS), or combined (pharmacological and mechanical) [1–3].

### Pharmacological prophylaxis [1–3]

Nowadays, pharmacological prophylaxis is essentially based on LMWH (bemiparin, dalteparin, enoxaparin, nadroparin, parnaparin or reviparin), although unfractionated heparin can be used in certain cases (1). As regards LMWH administration, although scientific studies identifying the optimal dose have not been published yet, it is advisable to give high doses (Table 3). Lower doses should, however, be considered in “fragile” patients (e.g., low body weight, renal insufficiency) (see information sheets provided with pharmaceuticals).

**Table 3:** Low-molecular-weight heparin (LMWH) products available in Italy

Active principle	Brand name	Dose and timing
Enoxaparin	Clexane^®^	4,000 IU 12 h before the operation, then 4,000 IU/day
Nadroparin	Fraxiparina^®^ Seleparina^®^	38 IU/kg 12 h before the operation and 12 h afterward, 38 IU/kg every 24 h over the next 3 days after surgery, then increasing the dose to 57 IU/kg/day
Dalteparin	Fragmin^®^	5,000 IU 8–12 h before the operation, then 5,000 IU/day Alternatively, 2,500 IU 1–2 h before surgery^a^ and 2,500 IU 8–12 after, then 5000 IU/day
Bemiparin	Ivor^®^	3,500 IU 6 h after surgery, then 3,500 IU/day Alternatively, 3,500 IU 2 h before surgery^a^, then 3,500 IU/day
Parnaparin	Fluxum^®^	4,250 IU anti-Xa 12 h before the operation, then 4,250 IU anti-Xa/day
Reviparin	Clivarina^®^	4,200 IU anti-Xa 12 h before the operation, then 4,200 IU anti-Xa/day

^a^Despite the manufacturer’s instructions, pre-operative administration is not advised for these specific patients by this working group

### Mechanical prophylaxis [1–3]

Mechanical prophylaxis is based on the use of elastic compression stockings (passive mechanical prophylaxis) and intermittent pneumatic pumps (active mechanical prophylaxis) [1, 3].

Passive mechanical prophylaxis (thigh–foot or knee–foot stockings) increases the efficacy of pharmacological prophylaxis, and should be employed (bilaterally if possible) until good mobility and autonomous ambulation are recovered. Care must also be taken to ensure stockings are applied correctly (i.e., not too tight/loose) by the nursing staff and/or the patients themselves. It should not, however, be prescribed if the patient has peripheral artery disease or diabetic neuropathy [1, 18].

Intermittent pneumatic compression pumps (sural or plantar) are highly efficacious and increase the action of anticoagulants, although their management has less compliance from nurses and patients [1].

Elective indications for mechanical prophylaxis are high risk of thrombosis accompanied by contraindications to pharmacological prophylaxis. In minor orthopaedic surgery, passive mechanical prophylaxis is often the only advisable prophylaxis in low-risk patients [1, 3].

### Timing of VTE prophylaxis

#### Arthroscopic surgery

Unlike hip or knee prosthetic surgery, where some studies and meta-analyses have been reported (with no significant differences between pre- and post-operative administration of pharmacological prophylaxis), there is no literature concerning the differences in terms of efficacy and safety of pre-surgical and post-surgical LMWH administration in arthroscopic surgery. Nonetheless, the working group reached the consensus that post-operative start would be wiser, even though in Italy the manufacturers recommend pre-operative administration (with the exception of bemiparin).

#### Non-arthroscopic orthopaedic surgery

Post-operative start of prophylaxis is also advisable in this case.

#### Emergency trauma surgery

For femoral neck fractures (see also the 2010 *Intersociety consensus statement* [2]), timing for an appropriate prophylaxis is strictly dependent on scheduled surgery:

- If surgery is performed immediately (within 24 h of the trauma), it is possible to start LMWH (12 h before or 12 h after surgery).
- If surgery is postponed, LMWH should be given early on, as soon as haemorrhagic contraindications (e.g., multiple traumas, severe head trauma) have been ruled out, then suspended 12 h prior to surgery and recommenced 12 h afterwards.
- Fondaparinux or new oral anticoagulants should not be used.

#### Non-surgical traumatology

In this case, antithrombotic prophylaxis (when indicated) should be started upon non-weight bearing and/or application of the cast or splint, etc.

### Duration of pharmacological prophylaxis

When indicated, the pharmacological prophylaxis should be administered for a minimum of 7 days. The duration of the prophylaxis should take into account the persistence of thromboembolic risk factors and the recovery of mobility and weight bearing (at least 10–20 kg).

## Special considerations in paediatric patients

A hotly debated issue in the working group meetings was pharmacological prophylaxis in paediatric patients (<18 years), particularly as little evidence is available in literature. In general, paediatric patients have a low risk of thrombosis, but this can be increased by other risk factors. In order to provide general indications for clinical application, the group analysed the data from the New York Stony Brook University Hospital trauma register [19]. No VTE was observed in any of over 1,000 trauma patients under 13 years of age who were not given prophylaxis. In over 1,000 13–17-year-old patients who were given prophylaxis, following the instructions of the individual surgeons, two episodes of VTE were recorded. More recent data, obtained in a traumatology setting [20, 21], confirm the low risk of VTE in paediatric patients.

Systematic pharmacological prophylaxis therefore finds no justification, but patients at greater risk need to be identified. No information on paediatric orthopaedic surgery was found in the literature, but general guidelines on hospitalized patients have recently been published by the Tuscany Regional Council [22].

The consensus on paediatric patients scheduled for major surgery was as follows:

- Pharmacological prophylaxis is not advisable in pre-pubertal patients, except in cases deemed to be at particular risk of thromboembolism.
- In pubertal and post-pubertal patients it is advisable to identify any risk factor and decide on individual case basis whether prophylaxis should be only mechanical or also pharmacological.

Among factors influencing such decision, particular attention should be paid to the presence of central vein catheterization and/or a previous history of VTE. It is also crucial taking into consideration obesity, family history of VTE at a young age (<50 years), parenteral nutrition, prolonged sedation, neuromuscular block, acute infection, the presence of neoplastic disease, major trauma and chronic illness.

## Classification of orthopaedic and trauma surgeries

Surgical procedures in orthopaedics and traumatology fall into four general categories: major orthopaedic surgery, minor orthopaedic surgery, major trauma surgery, and minor trauma surgery. Whether surgery is classified as major or minor will depend on a series of parameters, namely:

- The nature of the pathology
- Complexity and invasiveness of the treatment
- Technology required
- Comorbidity

## Prophylaxis in orthopaedic and trauma surgery

This section is dedicated to prophylactic approaches in various orthopaedic and trauma surgery procedures.

### Orthopaedics

#### Elective spinal surgery

VTE is a very rare but serious complication of spinal surgery. Analysis of the limited literature available reveals that the incidence of VTE appears to vary according to the presence of several factors as the invasiveness of surgery, the period of immobilization, the neurological damage and patient age [23–25]. The short period of time the patient is bed-bound following surgery is presumably one of the primary causes of low incidence of VTE. Posterior approach, the most common used, is associated with a very low incidence of VTE and any prophylaxis should, therefore, be chosen for its limited risk of complications. Indeed, pharmacological prophylaxis, which entails an increased risk of bleeding, could result in catastrophic compression of nervous system.

##### Type of prophylaxis

Mechanical prophylaxis (GCS, IPC) should be prescribed in cases of delayed recovery of ambulation, as its recognized beneficial effects are accompanied by an absence of correlated haemorrhagic complications. Pharmacological prophylaxis (LMWH), however, should be considered in cases of:

- Prolonged and/or complex surgery (e.g., combined anterior and posterior approaches)
- Patients with relevant VTE risk factors (see “Thrombotic risk”).

##### Timing and duration of prophylaxis

In the absence of evidence, the post-operative use of GCS is advisable until ambulation is recovered. Where pharmacological prophylaxis is deemed necessary, LMWH should be given after surgery and continued until normal ambulation is resumed.

#### Elective surgery of the pelvis and proximal femur (excluding prosthetic surgery)

Elective surgery of the pelvis and proximal femur (represented essentially by osteotomies and oncological surgery) has potentially a high risk of thromboembolism, and should, therefore, be considered as hip replacement surgery. Thus, albeit with no supporting evidence, pharmacological prophylaxis would be a wise precaution [1, 26, 27].

##### Type of prophylaxis

Pharmacological prophylaxis with LMWH or fondaparinux is advisable. Post-operative use of GCS, as an additional aid, might also be considered until ambulation is recovered.

##### Timing and duration of prophylaxis

It is advisable to start pharmacological prophylaxis in the post-operative period, and to continue until ambulation is resumed.

#### Elective knee surgery (excluding prosthetic surgery)

The need for VTE prophylaxis in patients undergoing elective knee surgery remains controversial, and it is necessary to distinguish between different types of procedure (major and minor) and the duration of the immobilization period [28–30].

##### Type of prophylaxis

Pharmacological prophylaxis with LMWH is advisable in major surgery. Pharmacological prophylaxis with LMWH is advisable in minor surgery only in the presence of additional risk factors linked to the procedure, such as, for example, the use of a tourniquet or non-weight bearing. Post-operative use of GCS, as an additional aid, may also be considered until ambulation is recovered.

##### Timing and duration of prophylaxis

Administration of pharmacological prophylaxis in the post-operative period is advised. The duration of prophylaxis should coincide with the period of immobilization of the limb or non-weight bearing. A minimum prophylaxis duration of 7 days is suggested.

#### Foot or ankle surgery

VTE risk in foot surgery has barely been studied. Nevertheless, the little available data from retrospective studies shows that the incidence of DVT ranges from 0.16 to 4 %, and that of PE from 0 to 0.15 % [31–35].

##### Type of prophylaxis

VTE prophylaxis is not advisable for patients with no risk factors. In those featuring general or surgery-related risk factors, such as, for instance, the use of a tourniquet, non-weight bearing, or ankle immobilization, pharmacological prophylaxis with LMWH should be considered.

##### Timing and duration of prophylaxis

Pharmacological prophylaxis in the post-operative period is advised. Prophylaxis duration should coincide with the period of immobilization of the limb or until weight bearing is resumed. A minimum prophylaxis of 7 days is advised when indicated.

#### Upper limb surgery

VTE is considered a rare complication in upper limb surgery and non-prosthetic surgery of the shoulder. In prosthetic surgery of the shoulder, a retrospective study reported a DVT incidence of 0.5 % [36, 37].

##### Type of prophylaxis

Pharmacological prophylaxis with LMWH is advised in prosthetic surgery of the shoulder. Pharmacological prophylaxis with LMWH should be considered in non-prosthetic surgery patients presenting risk factors.

##### Timing and duration of prophylaxis

It would be advisable to start pharmacological prophylaxis in the post-operative period. A minimum prophylaxis duration of 7 days is advised, and should accordingly be prolonged in patients confined to bed for an extended period.

#### Hip arthroscopy

VTE risk in hip arthroscopy has barely been evaluated, but retrospective studies appear to indicate an incidence ranging from 0 to 3.7 % [38–40] in the absence on prophylaxis.

##### Type of prophylaxis

VTE prophylaxis is not advisable for patients presenting no risk factors. Pharmacological prophylaxis with LMWH should be considered in patients featuring general or procedure-related risk factors, such as a prolonged surgery or non-weight bearing.

##### Timing and duration of prophylaxis

Administration of pharmacological prophylaxis in the post-operative period is advisable. Prophylaxis should be continued until the patient is able to bear weight, and, in any case, for at least 7 days. GCS may be advisable, as an additional aid, to be worn until the patient resumes ambulation.

#### Knee arthroscopy

This is the most studied type of arthroscopy as regards VTE risk [18, 27, 38, 41]. We distinguish between two types of knee arthroscopy, namely, major (i.e., ligament reconstruction) and minor (i.e., selective meniscectomy). A specific risk factor associated with knee arthroscopy is tourniquet use, especially if this is kept in place for longer than 60 min. Several studies have demonstrated the efficacy of LMWH in reducing the risk of VTE in this type of surgery without increasing the risk of haemorrhage.

##### Type of prophylaxis

Pharmacological prophylaxis with LMWH is always advisable in major surgery or in minor procedures if in presence of general or surgery-related risk factors, i.e., prolonged tourniquet application or non-weight bearing. Post-operative prescription of adjunctive GCS may also be considered until the patient resumes ambulation.

##### Timing and duration of prophylaxis

Pharmacological prophylaxis, when indicated, should be started postoperatively and its duration should coincide with limb immobilization or non-weight bearing. A minimum prophylaxis duration of 7 days is advised.

#### Ankle arthroscopy

Ankle arthroscopy surgery has been little analysed in terms of VTE risk [38]. Nevertheless, the incidence of VTE after ankle arthroscopy, extrapolated from a review of 15 studies (a total of 1,367 patients), appears to be 0 %.

##### Type of prophylaxis

VTE prophylaxis is, therefore, not advised in patients without risk factors. In patients with general or surgery-related risk factors, e.g., tourniquet application, ankle immobilization and non-weight bearing, pharmacological prophylaxis with LMWH should be considered.

##### Timing and duration of prophylaxis

Pharmacological prophylaxis is advised, when indicated, starting in the post-operative period. It should be administered for at least 7 days, and continued until mobilization and weight bearing.

#### Shoulder arthroscopy

Thromboembolic complications are very rare after shoulder arthroscopy, with a reported incidence of VTE of less than 0.01 % [36, 42].

##### Type of prophylaxis

VTE prophylaxis is not advisable in all patients, although those who feature risk factors may benefit from administration of LMWH.

##### Timing and duration of prophylaxis

Pharmacological prophylaxis, when indicated, should be started in the post-operative period, to be continued for at least 7 days.

#### Elbow or wrist arthroscopy

Neither elbow nor wrist arthroscopy have been studied as regards VTE risk and prophylaxis.

##### Type of prophylaxis

VTE prophylaxis is not advised.

### Trauma surgery

#### Amyelic vertebral fractures

There are two distinct types of treatment, surgical and conservative.

##### Surgical treatment

See the section on elective spinal surgery.

##### Conservative treatment

This is performed by means of casts or brace designed to immobilize the spine for 60–90 days. The patient may or may not be confined to bed for the first month.

##### Type of prophylaxis

Mechanical prophylaxis via GCS is advised.

Pharmacological prophylaxis may be advisable in cases of:

- Bed confinement
- Low mobility in patients featuring risk factors.

##### Timing and duration of prophylaxis

In cases of pharmacological prophylaxis, LMWH is advised, normally for 30 days, or less, if patient restores ambulation.

#### Upper limb fracture

VTE is considered a rare complication of upper limb or shoulder fracture.

##### Surgical treatment

See the section on elective surgery of the shoulder and upper limb.

##### Conservative treatment

The limb is immobilized by means of casts or specific braces.

##### Type of prophylaxis

Pharmacological prophylaxis (LMWH) is advisable only in cases of:

- Bed confinement
- Poorly mobile patients with risk factors
- Crushing injuries

##### Timing and duration of prophylaxis

LMWH is suggested, when pharmacological prophylaxis is required, normally for 30 days or until the patient restores mobility.

#### Pelvic or acetabulum fracture

Pelvic and acetabulum trauma presents a high risk of thromboembolism in both unstable fractures requiring surgery and stable fractures, due to the need for bed confinement [26, 27, 43–45]. In cases of multiple fractures, the high risk of VTE is not usually associated with an increase in haemorrhagic risk. In cases of multiple traumas, however, haemorrhagic risk needs to be considered greater than VTE risk, and pharmacological prophylaxis should be delayed until patient’s haemostatic conditions have been stabilized.

##### Surgical treatment

See the section on elective pelvic surgery.

##### Conservative treatment

Stable lesions that require no surgical treatment usually are treated with non-weight bearing periods of 3–5 weeks, and will, therefore, feature a high risk of VTE.

##### Type of prophylaxis

Pharmacological prophylaxis with LMWH is advised, and GCS should be considered as an additional aid until ambulation is resumed.

##### Timing and duration of prophylaxis

It is advisable to continue prophylaxis until ambulation is restored.

#### Lower limb fracture and traumatic lesions requiring immobilization

The reported incidence of VTE in these cases is somewhat variable (4.3–40 %). The incidence of total and proximal DVT in Achilles’ tendon rupture may reach 36 and 7 %, respectively. In patients with an immobilized lower limb, a meta-analysis performed on six randomized controlled trials, revealed a reduction in asymptomatic DVT from 17.1 to 9.6 % with LMWH pharmacological prophylaxis, without any associated increase in bleeding [28–30]. However, there is considerable controversy in literature regarding this issue [46–48]. This prompted the working group to formulate the following advice, which takes into account the known risk of VTE linked to ankle immobilization, the difficulty in precise risk stratification in an emergency situation, and the low risk of significant complications associated with pharmacological prophylaxis.

##### Surgical treatment

See the section on elective lower limb surgery.

##### Conservative treatment

Indications for conservative treatment with cast or brace are less than in the past but, in these cases, one or more joints may need to be immobilized.

##### Type of prophylaxis

Pharmacological prophylaxis with LMWH is advised in lower limb immobilization or complete non-weight bearing.

##### Timing and duration of prophylaxis

Pharmacological prophylaxis with LMWH is advised until patient is mobilized and ankle mobility and weight bearing are at least partially restored.

## Anaesthesia issues

The relationships and reciprocal influences on VTE prevention between anaesthesia and orthopaedic and trauma surgery have been well known for years. This brief summary aims to outline the key aspects of these complex interactions.

### Pro-coagulant effects of general anaesthesia

The literature, albeit somewhat dated, seems to suggest that the type of anaesthesia may influence the risk of VTE. General anaesthesia (GA) may contribute to DVT onset due to venous stasis through vasodilation and a consequent increase in venous capacitance and reduction in venous return, the latter being a further obstacle to the circulatory effects of positive pressure ventilation. Vasodilation can also damage the endothelium, thereby exposing the subendothelial layer, which, participating in coagulation cascade activation, promotes thrombus formation [49, 50].

### Anticoagulant effects of locoregional anaesthesia

Locoregional anaesthesia (LRA), whether neuraxial or peripheral, would appear to reduce the incidence of VTE, at least according to studies performed in vascular and major orthopaedic surgery on lower limbs [51, 52]. The mechanisms to explain this effect include rheological changes in the hyperkinetic blood flow in the lower limbs. Furthermore, epidural anaesthesia exerts a profibrinolytic action, which, however, does not seem to be clinically relevant, except at very high doses [53–56]. The effect on haemostasis is probably obtained through various mechanisms, including sympathetic afferent nerve blockade, a reduction in circulating catecholamines, and the pharmacological properties of the local anaesthetic systemically absorbed in small quantities [57–59]. All these combined effects cohoperate to a lower incidence of thrombosis in lower limb surgery performed under locoregional anaesthesia.

### Confounding factors

The above mentioned mechanisms of action are difficult to demonstrate, and, when evaluating the relevant studies, it is necessary to take into account the variability in surgical techniques, patient position and fluid management strategies employed, as well as the reduction in cardiac output, the choice of hypotensive anaesthesia, intra-operative hypovolaemia, blood loss and hypothermia, all factors that could play a role in the onset of DVT, irrespective of the anaesthetic approach used. Moreover, the majority of the reviewed studies involved patients who were not given the recommended pharmacological prophylaxis, or, in any case, were subjected to different prophylaxis regimens [60].

### Upper limb

Upper limb surgery can be performed with patient under either GA or LRA. LRA techniques involve local anaesthetic injection targeting the brachial plexus, at various levels, and therefore, a temporary blockade of the action potentials conducted by sensory and motor fibres, mainly corresponding to the anterior roots between C5 and C7. Positioning of a perineural catheter allows continuous perfusion of local anaesthetic to be maintained in the post-operative period. It also enables more efficacious antalgic control with respect to systemic analgesia, and possibly a better control of the stress reaction [61]. No statistically significant differences have been observed among different anaesthetic techniques for the incidence of upper limb DVT, although LRA is known to be associated with less pain after surgery and early mobilization, especially if continued in the post-operative period.

### Lower limb

Lower limb surgery as well can be performed with the aid of GA and LRA. In the latter, a distinction needs to be made between neuraxial LRA and peripheral nerve block. In terms of early dismissal from the operating theatre, with the introduction of new rapid-offset anaesthetics, there are no significant differences between LRA techniques. Whichever anaesthetic technique is employed, the use of a tourniquet must be considered as an independent risk factor, due both to the possibility of vascular endothelial damage and reperfusion-related phenomena [62].

### Epidural analgesia

Its positive effect seems to be linked not only to the prolonged efficacy and therefore to the benefits of epidural analgesia, but overall to a rapid post-operative patient mobilization, with a reduction of VTE risk [63].

### Risk/benefit ratio in the choice of type of anaesthesia

When choosing the anaesthetic technique it is crucial to accurately weigh up the risk/benefit ratio, taking into account the risk of thrombosis and/or haemorrhage after a careful evaluation of the variables involved: patient-related, proposed surgical technique, expected blood loss, the presence of pre-existing pathologies that can themselves increase the thromboembolic or haemorrhagic risk (cardiopathies featuring blood stasis and/or those that require prescription of drugs that interfere with coagulation, liver disease, nephropathies and blood diseases, etc.). Peripheral blockade should be considered a valid option in these cases [64]. A more detailed discussion of this issue can be found in the 2010 intersociety consensus statement [2].

## The role of general practitioners (GPs) in VTE prophylaxis continuation

General practitioners (GPs) are responsible for the care of patients in the community, and are, therefore, charged with guaranteeing the continuity of care of patients discharged from hospital.

The GP has an important role in VTE management. Minor orthopaedic and trauma patients are quickly discharged by hospitals frequently with a prescription of DVT prophylaxis.

Maximal VTE risk is in the first 2–3 weeks after trauma or surgery but the risk lasts for 2–3 months [1, 3]. This is a period where patients are mainly followed by a GP who is in first line in recognizing initial DVT-PE signs. Thus, a specific attention to VTE problems is, today more than ever, fundamental in the GP’s knowledge.

In fact, the GP is defined as the medical guarantor of patient health, starting from the basal fundamental actions necessary to keep as smooth as possible the process of homecare. The fundamental importance of GPs lies in the fact that they have a long-term relationship, not only with individual patients, but also with their close relatives, and they are, therefore, well informed as regards family, as well as personal history of illness, in addition to issues such as reliability, compliance, socioeconomic standing, and living arrangements. The GP is also seen as the ‘case manager’ in cases of domiciliary care.

In our specific context, the GP and the attending physician or surgeon must collaborate to ensure continuity of care before, during and after hospital treatment. The GP is, therefore, charged with providing the attending physician or surgeon with all the information they need to ensure that the patient can be treated with as few complications as possible. To this end, information technology tools are extremely helpful, particularly if the GP highlights relevant information in a patient’s records, thereby facilitating the triage procedure. Indeed, it is fundamental that in the case of scheduled hospitalization the GP is aware of the individual risk factors of the patient and records them appropriately so they are readily available for consultation upon admission. In this way, the attending physician or surgeon can weigh up the specific risks linked to the reasons for hospitalization alongside those presented by these patient-related factors. This will give them an accurate idea of the total risk the patient is likely to be exposed to, and help them choose the appropriate treatment strategy accordingly.

Once the patient is discharged from hospital, and therefore, re-entrusted to the care of the GP, the latter will need to monitor the patient’s progress and ensure that she/he adhere to the prescribed treatment, not only in terms of dosage and duration, but also in terms of behavioural compliance, as well as being on the look out for any delayed complications. This is especially true in the present hospital practice, where many elective surgeries are rapidly completed, sometimes even on an outpatient basis. Bearing in mind the key role of GPs, this intersociety consensus statement was drawn up to give them the best possible support in deciding whether or not to prescribe treatment in cases where there is no established risk/benefit ratio, and therefore, no clear guidelines. Although the same paucity of evidence also prevented us from making firm recommendations, having reviewed the literature and drawn on the combined experience of the working group participants, we are in a position to advise the clinician to consider whether such treatment may be necessary for the patient.

## Conclusions and future directions

This document represents the consensus of Italian experts in the field, drawn from the scientific state-of-the-art and available drug labels, as of the summer 2012, and will be made public by the five participant societies with different modalities (association journals and/or websites, symposia at national conferences, etc.).

It is also the authors’ intention to review the consensus statement on a regular basis, as and when new information comes to light. The FONDACAST study comparing the relative efficacies of 2.5 mg fondaparinux and nadroparin as regards VTE prevention in distal fracture of the lower limbs and Achilles’ tendon rupture has recently been concluded, although not yet published. If the preliminary results are confirmed, fondaparinux may become the drug of choice in this specific prophylaxis setting in the next future. Another novelty on the horizon is represented by the new oral anticoagulants currently being trialled. Although there are no scheduled trials of these drugs in VTE prevention in situations not involving hip and knee replacement surgery, the expected increased diffusion of new drugs for stroke prevention in atrial fibrillation will doubtlessly increase the number of patients under new oral anticoagulants (e.g., for atrial fibrillation) needing orthopaedic surgery.
